# Postoperative Catatonia in a Patient with Major Depression: A Case Report Following Orthognathic Surgery

**DOI:** 10.1192/j.eurpsy.2026.11039

**Published:** 2026-07-31

**Authors:** Y. S. Ramírez Ruz, M. Arenas Morín

**Affiliations:** Department of Mental Health, Nova Hospital Clinic, Monterrey, Mexico

## Abstract

**Introduction:**

Catatonia is a severe neuropsychiatric syndrome characterized by motor, behavioral and autonomic alterations. Its etiology varies. In the postoperative period, it is an unusual condition and is often confused with delirium, delaying diagnosis and increasing the risk of complications (Oldham et al., 2018).

**Objectives:**

To describe a case of catatonia as a rare complication in the surgical setting of a patient with a history of major depression, highlighting the importance of early diagnosis and favorable response to treatment with benzodiazepines.

**Methods:**

We present the case of a 60-year-old woman with untreated major depression who underwent orthognathic surgery for a complex dentofacial deformity. There were no intraoperative complications. On the second postoperative day, she presented with an episode of psychomotor agitation, followed by neurological deterioration (Glasgow 6). She was intubated and transferred to the intensive care unit, where she received continuous sedation (opioid, GABAergic anesthetic, and benzodiazepine). She was successfully extubated at 48 hours with recovery of awake status (Glasgow 11). However, 72 hours after extubation, her neurological status deteriorated again (Glasgow 8). She was evaluated by the neurology department, which ruled out metabolic, infectious, and neurological etiologies through cerebrospinal fluid analysis, Gram staining, cultures and PCR testing for bacteria, viruses, and fungi, as well as a cranial CT scan ruling out space-occupying, ischemic or hemorrhagic lesions.

**Results:**

On clinical evaluation, the patient exhibited symptoms such as hypoactivity, mutism, motor negativism, waxy flexibility, and gegenhalten. The score on the Bush-Francis Catatonia Scale was 20. Based on these findings, the diagnosis of catatonia was established, and treatment with lorazepam (2 mg every 8 hours) via nasogastric tube was initiated. Within 12 hours of initiating treatment, marked clinical improvement was observed with complete neurological recovery.

**Conclusions:**

Catatonia in the postoperative period represents a diagnostic challenge since its symptoms may simulate delirium or other neurological complications. In this case, the history of untreated major depression, added to the physiological stress of surgery, acted as a triggering factor. Recognizing the association of catatonia with mood disorders and considering it in the differential diagnosis is crucial to initiate effective treatment and improve the clinical prognosis of the patient.

**Disclosure of Interest:**

None Declared

